# Hydroxyl Radical Modification of Collagen Type II Increases Its Arthritogenicity and Immunogenicity

**DOI:** 10.1371/journal.pone.0031199

**Published:** 2012-02-03

**Authors:** Uzma Shahab, Saheem Ahmad, Moinuddin, Kiran Dixit, Safia Habib, Khursheed Alam, Asif Ali

**Affiliations:** Department of Biochemistry, Faculty of Medicine, J.N. Medical College, Aligarh Muslim University, Aligarh, India; National Institute of Environmental Health Sciences, United States of America

## Abstract

**Background:**

The oxidation of proteins by endogenously generated free radicals causes structural modifications in the molecules that lead to generation of neo-antigenic epitopes that have implications in various autoimmune disorders, including rheumatoid arthritis (RA). Collagen induced arthritis (CIA) in rodents (rats and mice) is an accepted experimental model for RA.

**Methodology/Principal Findings:**

Hydroxyl radicals were generated by the Fenton reaction. Collagen type II (CII) was modified by ^•^OH radical (CII-OH) and analysed by ultraviolet-visible (UV-VIS), fluorescence and circular dichroism (CD) spectroscopy. The immunogenicity of native and modified CII was checked in female Lewis rats and specificity of the induced antibodies was ascertained by enzyme linked immunosorbent assay (ELISA). The extent of CIA was evaluated by visual inspection. We also estimated the oxidative and inflammatory markers in the sera of immunized rats. A slight change in the triple helical structure of CII as well as fragmentation was observed after hydroxyl radical modification. The modified CII was found to be highly arthritogenic and immunogenic as compared to the native form. The CII-OH immunized rats exhibited increased oxidative stress and inflammation as compared to the CII immunized rats in the control group.

**Conclusions/Significance:**

Neo-antigenic epitopes were generated on ^•^OH modified CII which rendered it highly immunogenic and arthritogenic as compared to the unmodified form. Since the rodent CIA model shares many features with human RA, these results illuminate the role of free radicals in human RA.

## Introduction

Rheumatoid arthritis (RA) is a common human autoimmune disease characterized by the chronic inflammation of synovial joints and subsequent progressive and erosive destruction of the articular tissue [1]. Increased oxidative stress and/or defective antioxidant status contribute to the pathology of RA [2]. The pathogenesis of RA is associated with the formation of free radicals and proinflammatory cytokines at the site of inflammation. The inflammatory process develops in the tissue of the synovium where leukocytes are recruited, accumulate and become primary sources of reactive oxygen species (ROS). Various studies provide evidence for the involvement of free radicals and ROS that are produced endogenously during aerobic metabolism at the sites of chronic inflammation in the pathogenesis of RA [2]–[4]. Cells are normally protected from ROS-induced damage by a variety of scavenging proteins, enzymes and chemical compounds which constitute the endogenous antioxidant systems [5]. Both enzymatic and non-enzymatic antioxidant systems are impaired in RA, exposing the RA patients to oxidative stress and lipid peroxidation [6], [7]. Proinflammatory cytokines such as tumour necrosis factor-α (TNF-α), interleukin (IL)-1β and IL-6 are reported to be important mediators of disease progression [8]. We hypothesize that formation of hydroxyl radicals at the site of inflammation result in oxidative modification of collagen type II (CII), leading to the generation of neo-antigenic epitopes that initiate enhanced autoimmunity.

Collagen type II (CII), the principal component of human articular cartilage, is the most characterized autoantigen in RA [9]. Collagen-induced arthritis (CIA) in rodents is a widely studied model of RA. Compared to other antigen-defined models based on cartilage proteins, CIA has a short interval between immunization and disease manifestation. CIA in rats, as an experimental animal model of inflammatory polyarthritis, displays clinical and pathological features similar to those of human RA that are dependent on both humoral and cellular immunity to the immunizing antigen [10].

In the present study, commercially available CII was modified with ^•^OH radical generated by Fenton's reaction. Native and ^•^OH modified CII were characterized by UV-visible and fluorescence spectroscopy as well as far-UV circular dichroic spectropolarimetry and sodium dodecyl sulphate polyacrylamide gel electrophoresis (SDS-PAGE). The antigenicity of native and ^•^OH modified CII was probed in experimental rats. The arthritogenicity of both the conformers was established in terms of percent incidence and by giving scores to the rats' paw after visual inspection. This study also aimed to evaluate any impairment in the antioxidant system by assaying various oxidative stress markers in the immunized rats.

## Materials and Methods

### Ethics Statement

This study was approved by animal ethics committee of J.N. Medical College, AMU, Aligarh, India.

### Materials

All the reagents, including CII, were obtained from Sigma-Aldrich (St. Louis, MO USA) unless otherwise stated. Acetic acid and FeSO_4_ were from Qualigens, India. Hydrogen peroxide (H_2_O_2_) was procured from SRL, India. All other chemicals were of the highest grade available.

### Modification of collagen type II

Collagen type II (CII) from bovine nasal septum was modified with hydroxyl radical (^•^OH) generated by Fenton's reaction. CII was dissolved in 0.01 M acetic acid at a concentration of 2 mg/ml. The reaction mixture had 132 µg/ml CII, 0.0166 mM ferrous sulphate (FeSO_4_) and 0.33 mM hydrogen peroxide (H_2_O_2_). The reaction was run for 30 min at 37°C in a volume of 3 ml containing 0.01 M acetic acid. The controls were CII, CII+ FeSO_4_ and CII+ H_2_O_2_. All the controls were incubated for 30 min at 37°C to maintain identical experimental conditions. Modifications in CII were ascertained by UV-Spectral analysis, SDS-PAGE and circular dichroism.

### Immunization of rats/Induction of arthritis

Female Lewis rats 6–7 weeks old, with a mean weight of 175–200 g at the start of the experiment were used throughout the study. The rats were maintained under climate-controlled conditions in a 12 hr light and dark cycle. The animals were fed with standard rodent chow and were given normal drinking water. Rats were subdivided into the following groups: (1) control (*n* = 6); (2) CII (*n* = 6); (3) CII-OH (*n* = 6).

The rats were immunized as described previously [11]. Briefly, CII and CII-OH (2 mg/ml each) were emulsified with Freund's complete adjuvant at 1∶1 v/v ratio. The rats were immunized intradermally at the base of tail with 250 µl emulsion; hence each rat received 250 µg antigen. Rats belonging to the CII group were challenged again 10 days later with an antigen preparation containing Freund's incomplete adjuvant.

### Arthritis evaluation

The arthritis development was monitored by visual inspection of paws from day 7 post immunization (p.i.) for CII-OH group and from day 17 p.i. for CII group (as there were no signs of arthritis in the CII group after the first dose) until the end of the experiment (day 30 p.i.). The severity of arthritis in each paw was quantified daily by clinical score measurement [12] from 0 to 4. The scoring was based on the degree of periarticular erythema and edema as well as deformity of the joints. The scores are given as follows: 0- no macroscopic signs of arthritis (erythema and swelling); 1- Erythema and mild swelling confined to the tarsals or ankle joint; 2- Erythema and mild swelling extending from the ankle to the tarsals; 3- Erythema and moderate swelling extending from the ankle to metatarsal joints; 4- Erythema and severe swelling encompassing the ankle, foot and digits, or ankylosis of the limb. The maximum score for each rat was 16.

### Sera collection

Blood samples were collected during the study by cardiac puncture on day 7 p.i. from CII-OH group and on day 17 p.i. from CII group. On day 30 p.i. blood samples were again withdrawn from all the groups. Sera were separated by centrifugation for 10 min at 3000 rpm and aliquots were stored at −20°C.

### Detection of anti-CII and anti-CII-OH serum antibodies

Antibodies were detected by ELISA as described earlier [13]. The specificity of the antibodies was determined by competitive binding assay [14].

### Isolation of IgG

Immunoglobulin G was isolated from immune sera on a 2.5 ml Protein A-Agarose pre-packed column [11]. The homogeneity of isolated IgG was verified on SDS-PAGE.

### Oxidative stress evaluation

Serum malondialdehyde (MDA) content was determined by the method of K. Yagi [15]. Superoxide dismutase (SOD) was determined by the pyragallol auto-oxidation method [16] and glutathione (GSH) by Ellman's method [17], with slight modifications in each. Catalase activity was evaluated according to Abei's method [18]. Serum carbonyl content was analyzed by the method of Levine *et al.* [19], with slight modifications. Glutathione peroxidase (GPx) activity was checked by the method of Flohe *et al.* [20]. Protein concentration in serum samples was estimated by the Lowry method [21].

### Quantification of serum inflammatory markers

Tumour necrosis factor-α (TNF-α) and interferon-γ (IFN-γ) concentrations were determined in rat sera by rat TNF-α and IFN-γ ELISA kits (Thermo Fischer Scientific, USA) according to the manufacturer's instructions. Absorbance was measured on an ELISA plate reader at 450 nm and 550 nm. The 550 nm values were subtracted from the 450 nm values to correct the optical imperfections in the microplate.

### Statistical analysis

Data are expressed as means ± SD. The difference between the means of the three groups was evaluated with 1-way ANOVA and considered significant at *P*<0.05.

## Results

**Figure 1:**
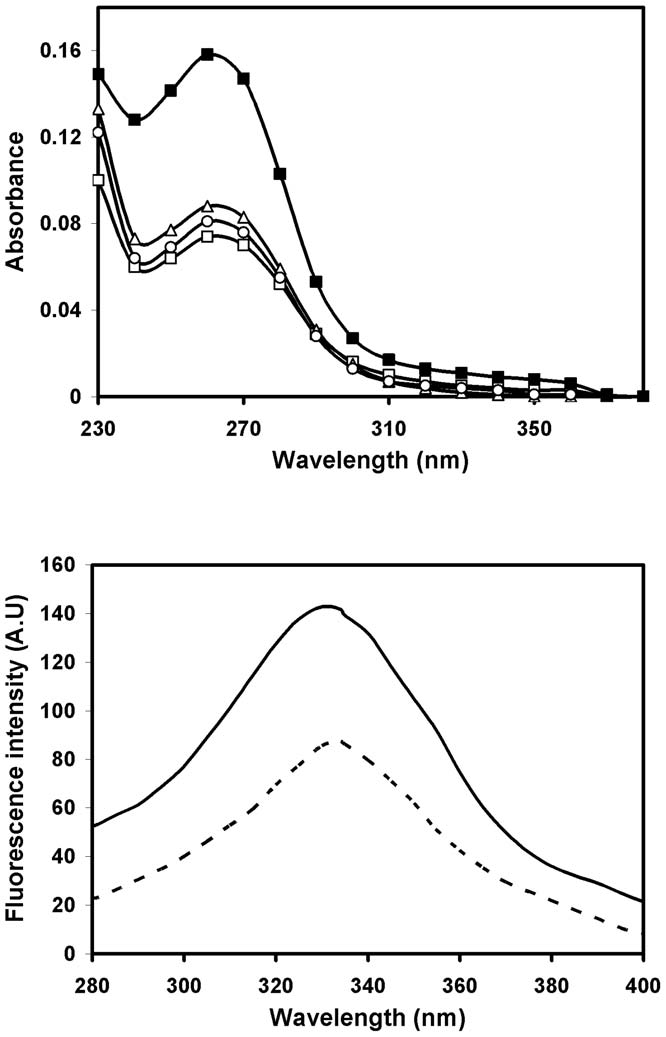
(a) UV absorption spectral profile of CII (□), CII+H_2_O_2_ (○), CII+FeSO_4_ (▵) and ^•^OH-modified CII (▪). (b) Fluorescence emission spectra of native CII(—) and ^•^OH-modified CII (---), Excitation wavelength was 265 nm.

The native CII was modified by ^•^OH radical generated by Fenton's reaction in the presence of H_2_O_2_ and FeSO_4_. Structural changes in the modified protein were analysed by UV absorption and CD spectroscopy. The native form of collagen type-II gave a characteristic peak at 263 nm in UV-spectrum while the ^•^OH modified CII (CII-OH) exhibited a significant increase of 52.8% in absorbance (Figure 1a). The corresponding controls, CII-H_2_O_2_ and CII-FeSO_4_, did not show any appreciable increase in absorbance when compared to the native CII.

Fluorescence emission profiles of CII excited at different wavelengths were recorded to determine the emission wavelength for maximum intensity (data not shown). Maximum emission was obtained at 324 nm after excitation at 265 nm. When CII-OH was excited at the same wavelength, a loss of 38.3% in fluorescence intensity was observed (Figure 1b).

**Figure 2:**
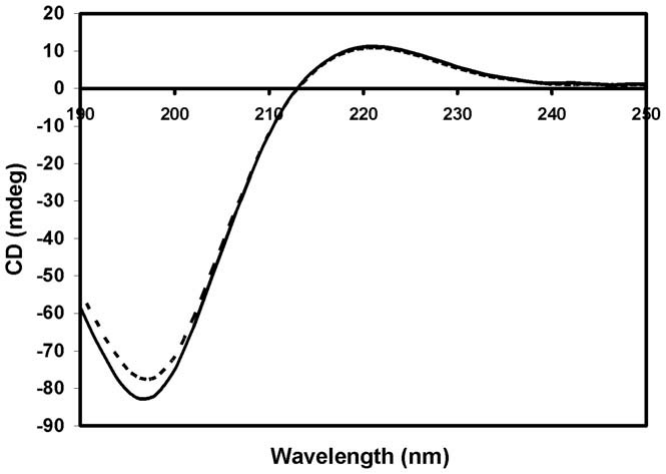
Far UV circular dichroic spectra of native (—) and ^•^OH- modified CII (- - -).

The circular dichroic (CD) spectra of protein solutions provide information about the secondary structure of proteins. Far UV-CD spectrum of native and modified CII exhibited a maxima at 221 nm and a minima at 197 nm, which are characteristic features of the collagen triple helix [22]. Upon ^•^OH oxidation, the positive CD signal at 221 nm remained unaltered at 11.307 mdeg in the far UV-CD spectrum. However, the negative signal at 197 nm shifted from -82.6 to -77.5 mdeg (Fig. 2). The secondary structure of CII was disrupted with a sharp decrease in CD signal resulting from the partial unfolding of CII. This change in the secondary structure of CII reflected certain conformational changes in the protein upon modification by ^•^OH.

**Figure 3:**
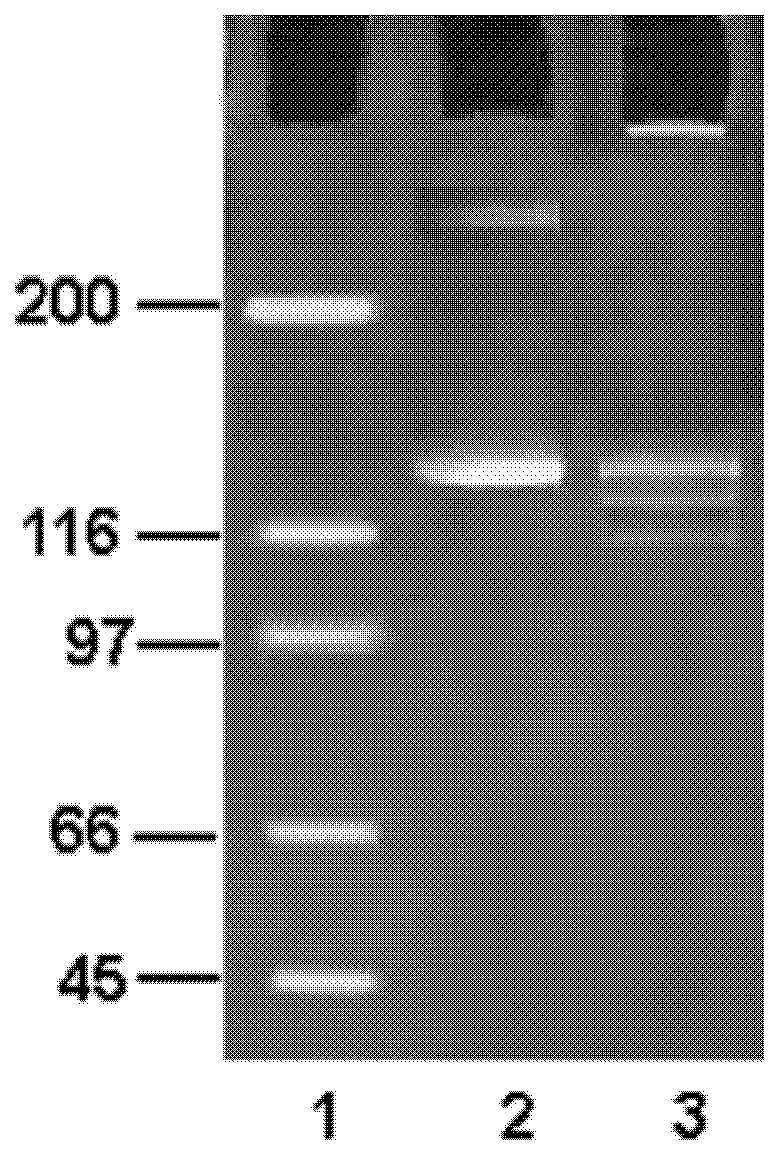
Denaturing SDS-PAGE of native and ^•^OH- modified CII. Equal amounts of protein (8 µg) were boiled with loading buffer containing β-mercaptoethanol for 3 minutes before loading on the gel. Lane 2 & 3 show native and modified CII respectively while lane 1 had protein marker.

In SDS-PAGE, native CII showed a major electrophoretic band that migrated just above the 116-kD band of protein marker, as well as traces of higher molecular weight aggregates at approximately 260 kD (Figure 3). The major band at approximately 130 kD corresponded to the constituent α-chains of CII. The apparent molecular mass of 130 kD reflected the well-known slow migration of collagen α-chains relative to globular protein markers. The exposure of CII to hydroxyl radical caused an extensive loss of the polypeptide backbone resulting in faint bands of low molecular weight with a faster migration, probably due to the ^•^OH mediated fragmentation of protein.

**Figure 4:**
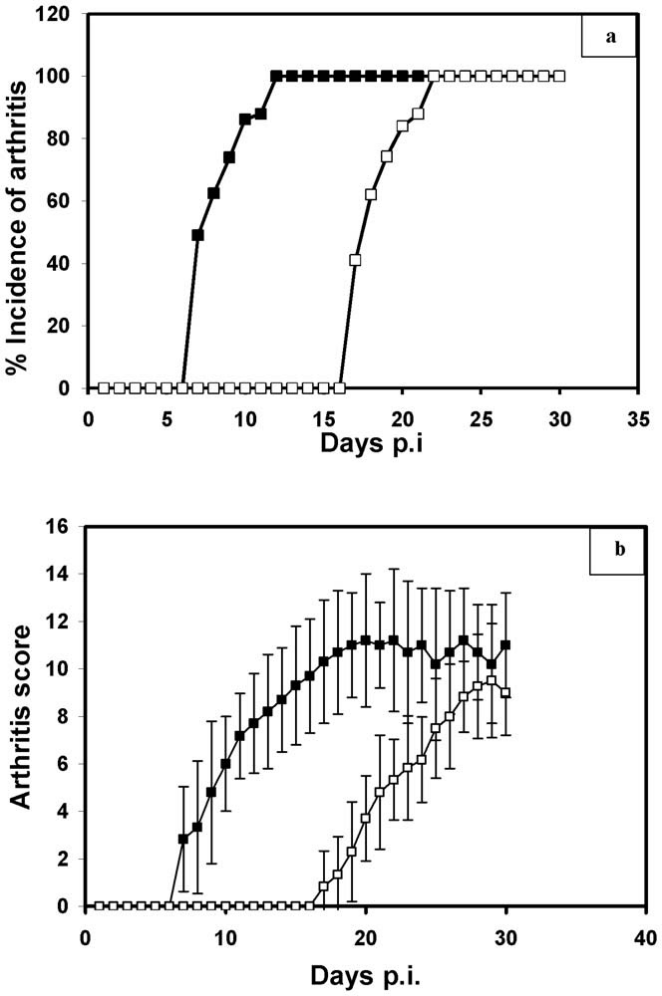
(a) Incidence of arthritis induced by native (□) and ^•^OH- modified CII (▪). In rats immunized with CII–OH, clinical arthritis started to develop by day 7 post-immunization (p.i.) and reached to 100% incidence by day 12 p.i. In contrast, rats immunized with native CII, developed arthritis by day 17 p.i. that reached 100% by day 22 p.i. (b) Severity of arthritis induced by native (□) and ^•^OH modified (▪) CII. Rats immunized with CII–OH showed an average score of 7.7 by day 12 p.i. which continued to rise until the end of the experiment when the average score was found to be 11. Whereas, rats immunized with native CII showed an average score of 5.3 at day 22 p.i. which increased to 9 at the end of experiment.

After immunization with CII and CII-OH, rats were inspected visually. Seven days p.i., animals began to show evidence of clinical inflammation in one or both hind paws in the CII-OH group. A 250 µg dosage of CII-OH resulted in chronic inflammatory arthritis that reached 100% by day 12 p.i., whereas the same dose of native CII had no effect within the same time period. For the native CII, a booster dose had to be given on day 10 p.i. and inflammatory sign was observed on day 17 p.i. with 100% incidence on day 22 p.i., (Figure 4a). This indicates that ^•^OH modification of CII caused an early onset of arthritis with increased arthritogenicity. By day 12 p.i., an average score of 7.7 was reached which subsequently increased to 11 by day 30 p.i. for the group receiving CII-OH. Whereas, for the group receiving the native analogue of protein, the average score on day 22 p.i. was 5.3 which increased to 9 on day 30 p.i. (Figure 4b), indicating a slow and less severe progression of disease. To probe adjuvant induced arthritis (AA), we also immunized one group of rats with CFA with the booster dose of IFA given on day 8. No signs of inflammation were observed in this group, ruling out the AA in our study.

**Figure 5:**
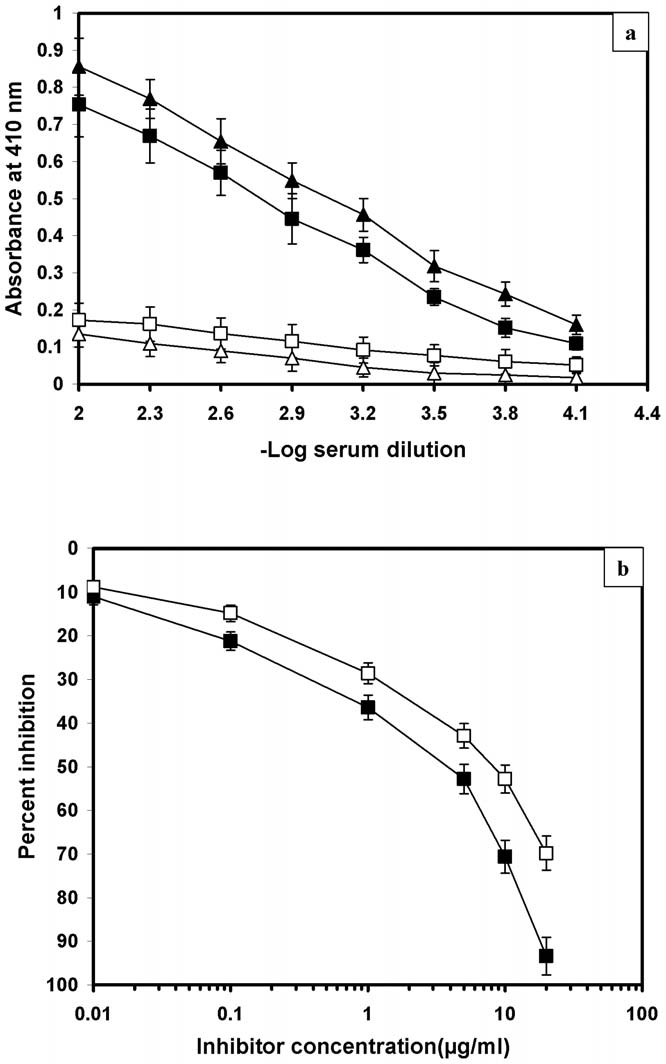
(a) Levels of induced antibodies against ^•^OH-modified CII and native CII. (▴) and (▵) respectively represent immune and preimmune sera for ^•^OH-modified collagen (CII-OH). While, (▪) and (□) represent immune and preimmune sera for native collagen (CII). ELISA plates were coated with CII-OH and native CII respectively (10 µg/ml). Each assay was done in triplicate. (b) Competitive-inhibition ELISA of anti-CII-OH IgG binding to CII-OH. The inhibitors were native CII (□), CII-OH (▪). Microtitre plates were coated with CII-OH (10 µg/ml).

Immunization with ^•^OH-modified CII in rats induced high titre antibodies (≥1∶12800), whereas native CII elicited a moderate response with antibody titre of ≥1∶6400. Preimmune serum showed negligible binding with the respective immunogens (Figure 5a). IgG was purified from preimmune and immune rat antiserum by affinity chromatography on a Protein A-Agarose column. The purified IgG eluted in a single symmetrical peak and the homogeneity was confirmed by a single band in SDS-PAGE (data not shown). The antigenic specificity of the induced anti-CII-OH antibodies was evaluated by competitive inhibition assay. A maximum of 93.4% inhibition of the anti-CII-OH IgG activity was observed when immunogen was used as an inhibitor. Native CII caused only 63.8% inhibition when used as an inhibitor (Figure 5b).

The oxidative stress status was checked in the immunized animals by measuring various antioxidant markers (Table 1). Glutathione (GSH) concentration was evaluated to estimate endogenous defences against oxygen metabolites. A marked decrease in GSH concentration was observed in the CII-OH group rats as compared to the CII group in relation to the control values. Superoxide dismutase (SOD) activity was determined to estimate endogenous defences against superoxide anion. A significant decrease in this antioxidant was seen in CII-OH group rats, as compared to the CII group. Serum glutathione peroxidase (GPx) and catalase (CAT) activities were assessed to check H_2_O_2_ mediated damage. A decline in the activity of these enzymes was observed in the collagen induced arthritic animals, which was more pronounced in CII-OH group rats. Protein carbonyl groups serve as a biomarker of oxidative stress, since protein oxidation typically results in increased carbonyl contents. These were found to be maximum in the CII-OH group. Malondialdehyde was assayed to estimate free-radical damage to biological membranes. Low levels were recorded in the control group at the end of the experiment (day 30 p.i.); these values were considered normal. In contrast, a significant increase in malondialdehyde level was found in the sera of CII-OH group rats compared to CII group rats.

**Table 1:** Clinical features in collagen induced arthritis rat sera.

Parameters	Control	CII*	CII-OH**
SOD (U/ml)	46.83±2.1	32.53±2.3	26.42±2.5
GPx (U/mL)	0.041±0.002	0.037±.003	0.031±0.002
CAT (U/mL)	1072±13	784±11.2	638±11.8
GSH (nmol/ml)	17.42±1.7	12.58±1.5	10.3±1.4
Carbonyl content (nmol/ml)	154±2.8	221±3.2	254±3.7
MDA (nmol/ml)	2.34±0.4	3.02±0.5	3.8±0.5
IFN-γ (pg/ml)	74.8±2.5	224.5±7.4	368.2±8.4
TNF-α (pg/ml)	38.4±2.3	380.3±8.9	428.6±11.8

All the Experiments were repeated thrice for reproducibility.

The values represented here indicate ±S.D for six rats in each group.

*P<0.01 versus control;

**P<0.05 versus CII.

TNF-α and IFN-γ levels were assayed in the serum of each group of rats, at the end of the experiment. In the control group, the average level of TNF-α was 38.4±2.3 pg/ml. A marked increase in TNF-α concentration was found in the serum of collagen induced arthritic rats with the maximum value of 428.6±11.8 pg/ml in CII-OH group. In the CII group, the average value recorded was 380.3±8.9 pg/ml. A similar pattern was seen in case of IFN-γ. The highest concentration of the cytokine (368.2 pg/ml) was observed in CII-OH group rats; while for the CII group and control, the observed values were 224.5±7.4 and 74.8±2.5 pg/ml respectively (Table 1). The increase in the values of TNF-α and IFN-γ in the CII-OH group was significant with a p value of <0.01 versus control and <0.05 versus CII.

## Discussion

Free radicals have long been implicated as mediators of tissue damage in RA patients [23]. Phagocytes trigger a respiratory burst characterized by increased oxygen consumption, increased anaerobic glycolysis and generation of oxygen radicals. An excessive production of free radicals can lower or even destroy defences against oxidative stress [24]. When endogenous antioxidant defences are overcome, free radicals induce impairment and destruction of the affected joint constituents such as synovial fluid, cartilage and other articular constituents, including collagen type II. Antibodies to native CII have been reported in RA since 1970s [25]. However, the clinical significance of this observation has been unclear as the incidence of anti-native CII antibodies in patients with RA varies widely [26], [27]. Moreover, anti-native CII antibodies have been found in other diseases as well as in healthy controls [28].

In the present study, CII was exposed to the hydroxyl radical, a highly toxic species and a powerful oxidizing agent [29] that leads to the formation of neo-antigens that in turn are thought to initiate autoimmune response.

The ^•^OH radical was generated *in vitro* by Fenton's reaction. Incubation of commercially available CII with the ^•^OH radical resulted in extensive damage to the protein as evident from UV-visible, fluorescence and CD spectral studies, as well as by the band pattern in SDS-PAGE. In the UV absorption spectral studies, 52.8% hyperchromicity observed in the modified CII as compared to its native analogue can be attributed to the exposure of chromophoric groups of CII as a result of modification. A loss of 38.3% in fluorescence intensity was also observed in CII-OH. These spectroscopical changes indicate substantial structural perturbations in the CII molecule as a consequence of ^•^OH modification.

Collagen is an optically active protein that adopts a polyproline II-like helical conformation. The unique CD spectrum of collagen is characterized by a small positive band at 221 nm and a large negative band at 197 nm. [30]. The maxima of the two bands is a measure of the triple helical content of the given sample [31]. After modification with ^•^OH, the characteristic peak of CII at 221 nm was retained, suggesting no change in the packing of helices and protein conformation. However, a slight decrease in the negative CD value indicated the conversion of some of the triple helical content into random coils. This is less severe than modification with HOCl that leads to a complete loss of the triple helical content [32].

In SDS-PAGE, modified collagen showed faster mobility compared to the native analogue, suggesting fragmentation of the protein resulting in small molecular weight peptides.

In this study we have chosen to use CIA, an animal model that is characterized by peripheral joint lesions. We have demonstrated a substantial increase in immunogenicity and arthritogenicity of CII upon ^•^OH modification. Immunization with denatured CII α-chains in an earlier study, induced only a weak antibody response and was not arthritogenic [33], suggesting that the antibody response to CII is predominantly directed towards the conformational triple-helical structure.

Immunization with hydroxyl radical modified collagen (CII-OH) resulted in an early and more severe arthritis as compared to native CII. This shows that structural perturbations in the CII upon modification by ^•^OH have rendered it highly immunogenic.

In the CIA model, the triggering of auto-reactive B cells by CII is undoubtedly an important pathogenic factor during the acute phase of the disease. B cell-deficient mice are completely resistant to CIA [34] and arthritis can be passively transferred with immune sera [35]. However, the development of arthritis has not been perfectly correlated with serum titres of antibodies against CII because high titres of antibodies do not always lead to severe arthritis [36]. In our studies, enhanced arthritogenicity of hydroxyl radical modified CII was coupled to its increased immunogenicity in the experimental rats. The native CII was moderately immunogenic with relatively low arthritogenicity compared to the ^•^OH modified CII.

In the present study, a change in the levels of oxidative stress markers and physiological antioxidants was observed in the animals with CIA as compared to the healthy controls. The higher amount of malondialdehyde found in the CII-OH group, as compared to CII, at day 30 p.i. is indicative of increased lipid peroxidation in CIA rats. It is pertinent to mention here that lipid peroxidation has been reported as a critical mechanism of injury that occurs during RA [37].

The production of oxygen free radicals with the onset and progression of arthritis in the articular cartilage leads to decreased GSH and SOD levels as a consequence of their consumption during oxidative stress and cellular lysis [38]. For similar reasons, there is a decline in CAT and GPx specific activity. This decrease favours increased cellular damage by free radicals. The decreased antioxidants levels in the CII-OH group can be described as a result of the high intensity of arthritis in that group.

The carbonyl content analysis is a general assay of oxidative protein damage. Several reactive species including ^•^OH, oxidize amino acid residues in proteins to form products with carbonyl groups that can be measured after reaction with 2–4 dinitro phenyl hydrazine (DNPH). Carbonyl content was measured in the serum samples of rats belonging to different groups. The highest value was recorded in the CII-OH group followed by the CII group, whereas the control group had the least carbonyl content. This shows maximum oxidative protein damage in CII-OH group that can be correlated to the severe arthritis in the CII-OH group rats.

Several areas of investigation have indirectly implicated TNF-α and IFN-γ as contributors to cellular damage in CIA. IFN-γ is secreted by T-cells whereas TNF-α is secreted by several types of cells, including lymphocytes and macrophages. The high levels of these cytokines have been interpreted as a progression of cartilage cell injury [39]. Elevated levels of serum inflammatory markers in the CII-OH group were indicative of severe inflammation in the rats immunized with ^•^OH modified collagen.

In conclusion, we propose that the oxidative modification of CII, possibly in combination with proteolysis, creates a CII autoantigen. It is possible that in the inflamed joints, abnormally high fluxes of reactive oxygen species give rise to chemical reactions that modify CII. It has been suggested that a breakdown of tolerance occurs because antibodies against modified self protein are promiscuous and bind both the modified and unmodified self antigen. This process is commonly characterized as epitope spreading. Our study extends this hypothesis, suggesting that modifications of CII may contribute to the vicious cycle of chronicity by providing additional epitopes to which the immune system is intolerant, resulting in the stimulation of the immune response against self antigens. Convincing evidence has been presented on the role of oxygen free radicals in the pathogenesis of RA. Superoxide produced in synovial fluid could generate •OH radical via Fenton reaction at the inflammation site [40]. This ^•^OH can damage cellular elements in the cartilage and extra cellular matrix components, including proteoglycan and collagen type II. Since the rat CIA model shares many features with human RA, our results may help in explaining certain aspects of the human RA, especially the role of free radicals. We hope our findings would pave the way for further studies in this direction.
